# Inhibition of FGFR2 Signaling by Cynaroside Attenuates Liver Fibrosis

**DOI:** 10.3390/ph16040548

**Published:** 2023-04-06

**Authors:** Qilin Meng, Lin Luo, Minghua Lei, Zhiqi Chen, Yuanmeng Sun, Xue Chen, Zhaodong Zhai, Yibo Zhang, Jieqiong Cao, Zijian Su, Fu Li, Jingsheng Li, An Hong, Xiaojia Chen

**Affiliations:** 1 Department of Cell Biology, College of Life Science and Technology, Jinan University, Guangzhou 510632, China; 2 National Engineering Research Center of Genetic Medicine, Guangzhou 510632, China; 3 Guangdong Province Key Laboratory of Bioengineering Medicine, Guangzhou 510632, China; 4 Guangdong Provincial Biotechnology Drug & Engineering Technology Research Center, Guangzhou 510632, China

**Keywords:** liver fibrosis, collagen accumulation, FGFR2, cynaroside, hepatic stellate cells

## Abstract

Liver fibrosis represents a significant health hazard with a high morbidity rate and an increased risk of liver cancer. Targeting overactivated Fibroblast growth factor receptor 2 (FGFR2) is a promising strategy to counteract collagen accumulation during liver fibrosis. However, there is a shortage of drugs to specifically block the activation of FGFR2 in liver fibrosis patients. Data mining, cell validation, and animal studies showed a positive correlation between FGFR2 overexpression and liver fibrosis development. Novel FGFR2 inhibitors were screened using a microarray-based high-throughput binding analysis. The effectiveness of each candidate was validated through simulated docking, binding affinity verification, single-point mutation validation, and in vitro kinase inhibition measurements to demonstrate the ability of each inhibitor to block the catalytic pocket and reverse FGFR2 overactivation. A specific FGFR2 inhibitor, cynaroside (CYN, also known as luteoloside), was screened based on the finding that FGFR2 promotes hepatic stellate cell (HSC) activation and collagen secretion in hepatocytes. The results from cellular assays showed that CYN can inhibit FGFR2 hyperactivation resulting from its overexpression and excessive basic fibroblast growth factor (bFGF), reducing HSC activation and collagen secretion in hepatocytes. Animal experiments on a carbon tetrachloride (CCl_4_) mouse model and a nonalcoholic steatohepatitis mouse model indicate that CYN treatment reduces liver fibrosis during fibrosis formation. These findings suggest that CYN prevents liver fibrosis formation at the cell level and in mouse models.

## 1. Introduction

Liver fibrosis is a serious condition that can lead to significant rates of morbidity and mortality [1]. It is associated with an increased risk of liver-related complications, such as cirrhosis [2] and liver failure [3,4], as well as an increased risk of cardiovascular disease [5]. Notably, studies have shown that advanced liver fibrosis is a significant risk factor for the development and recurrence of liver cancer and that early treatment of fibrosis may help to prevent advancement to liver cancer [6,7].

Liver fibrosis is a normal physiological mechanism associated with liver repair, but when excessive and persistent, it can lead to cirrhosis and liver failure [3,8]. Studies have shown that hepatic stellate cell (HSC) activation and the subsequent secretion of collagen lead to excessive accumulation of the extracellular matrix (ECM) in the liver, leading to liver fibrosis [9]. After HSC activation, other hepatocytes, such as hepatic sinusoidal endothelial cells and hepatic myofibroblasts, are involved in collagen accumulation [10]. Collagen secretion is a decisive process in fibrogenesis and is regulated by multiple signals [11]. In recent years, a growing amount of research has focused on the molecular mechanisms underlying collagen accumulation in liver fibrosis, including the roles of cytokines, growth factors, and signaling pathways, such as transforming growth factor (TGF)-β [12,13], MAPK [14,15], and Wnt [14,16]. These studies have shed light on possible treatments for liver fibrosis.

Fibroblast growth factor receptor 2 (FGFR2) is a tyrosine kinase receptor that activates MAPK and Wnt signaling and plays a crucial role in the development of liver fibrosis. Studies have demonstrated that FGFR2 is overexpressed in activated HSCs [17,18]. Moreover, it has also been reported that FGFR2 overexpression can trigger the activation of HSCs [17,19]. This activation results in the secretion of ECM proteins as well as increased migration and proliferation of HSCs [17,20]. In addition, the genetic knockdown of FGFR2 has been shown to reduce liver fibrosis in various animal models. It was observed that FGFR2 knockdown significantly reduced the expression of collagen I and TGF-β1, key markers of liver fibrosis [21]. Overall, these studies suggest that FGFR2 plays a crucial role in the development of liver fibrosis, and the targeting of FGFR2 using specific inhibitors may be a potential therapeutic strategy for treating liver fibrosis.

Based on our data mining of the transcriptome sequences of human and murine liver fibrosis, we discovered a correlation between FGFR2 overexpression and liver fibrosis. In our study, we demonstrated that excessive activation of FGFR2 can stimulate HSC cell activation and increase sensitivity to TGF-β in both HSC cells and liver cells. Conversely, FGFR2 knockdown can hinder TGF-β-induced activation of HSC cells and collagen protein secretion in liver cells. This prompted us to screen for a small molecule inhibitor candidate that can specifically bind to the intracellular domain of FGFR2 via a high-throughput affinity analysis. The candidate binds and inactivates the kinase domain of FGFR2, thereby suppressing downstream signal activation and reducing multiple fibrosis markers. Our results from carbon-tetrachloride-induced and high-fat-diet-induced fibrosis models indicate that this candidate possesses the ability to resist liver fibrosis development. It could thus be used as a therapeutic approach for clinical treatment.

## 2. Results

### 2.1. High Expression of FGFR2 Coincides with Liver Fibrosis

**Figure 1:**
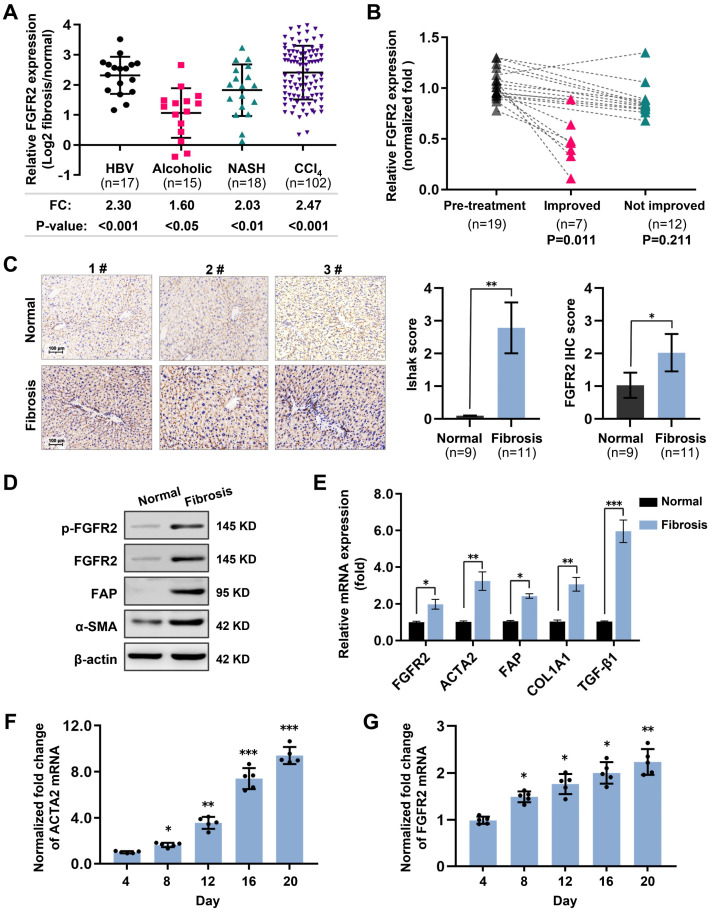
High expression of fibroblast growth factor receptor 2 (FGFR2) coincides with liver fibrosis. (**A**) The expression of FGFR2 in fibrotic liver and normal liver tissues was evaluated through data mining of human samples affected by Hepatitis B infection (GSE38941), alcohol abuse (GSE28619), and nonalcoholic steatohepatitis (GSE48452), as well as mouse samples induced with carbon tetrachloride (CCl_4_) (GSE152329, GSE98577). The differential fold expression was determined by comparing the expression of FGFR2 in the fibrotic liver and normal liver tissues. (**B**) A comparison of changes in FGFR2 expression in liver fibrosis patients in remission (improved) or not in remission (not improved) with mining data of GSE175448. (**C**) The correlation between FGFR2 expression and the liver fibrosis score (Ishak score) was analyzed by comparing FGFR2 expression in three pairs of liver fibrotic tissues and normal liver tissues through immunohistochemistry. (**D**) FGFR2 expression in liver fibrosis tissues and normal liver tissues was verified by Western blot and (**E**) q-PCR analyses. (**E**) Liver tissues from mice treated with CCl_4_ for different durations were used to determine the expression of the liver fibrosis markers Actin Alpha 2 (ACTA2) and FGFR2 by q-PCR and the trends of their expression with increasing days of induction. (**F**) The expression of ACTA2 and (**G**) FGFR2 was determined by q-PCR in liver tissues from mice treated with CCl_4_ for varying durations. The statistical significance was determined based on the *p*-value. The results are marked as significant “*” when *p* < 0.05, “**” when *p* < 0.01, “***” when *p* < 0.001, and not significant (ns) if *p* ≥ 0.05.

To elucidate the correlation between FGFR2 and liver fibrosis, this study first conducted data mining on the National Cancer for Biotechnology Information Gene Expression Omnibus (GEO) database. The expression of FGFR2 was found to be elevated in liver fibrosis patients with backgrounds of Hepatitis B infection (2.30-fold), alcohol abuse (1.60-fold), and non-alcoholic steatohepatitis (NASH, 2.03-fold) through a comparison with normal individuals. FGFR2 was also upregulated by 2.47-fold in the livers of mice with CCl_4_-induced liver fibrosis (Figure 1A). Furthermore, the analysis of FGFR2 expression during liver fibrosis regression revealed that the expression was decreased in patients whose fibrosis was in remission but unchanged in those who failed to be cured (Figure 1B). The upregulation of FGFR2 in liver fibrosis patients was confirmed by various methods, including immunohistochemistry (IHC) (Figure 1C), Western blot quantification (Figure 1D), and reverse transcription quantitative real-time PCR (RT-qPCR) quantification (Figure 1E) on liver tissue samples collected from clinical volunteers. In the CCl_4_-induced mouse fibrosis model, the expression of FGFR2 was positively correlated with the expression of Actin Alpha 2 (ACTA2), an indicator of fibrosis, as the number of days of induction increased (Figure 1F,G). These results suggest that FGFR2 expression is positively correlated with liver fibrosis progression and tends to increase as the degree of liver fibrosis increases.

### 2.2. FGFR2 Drives the Process of Liver Fibrosis

**Figure 2:**
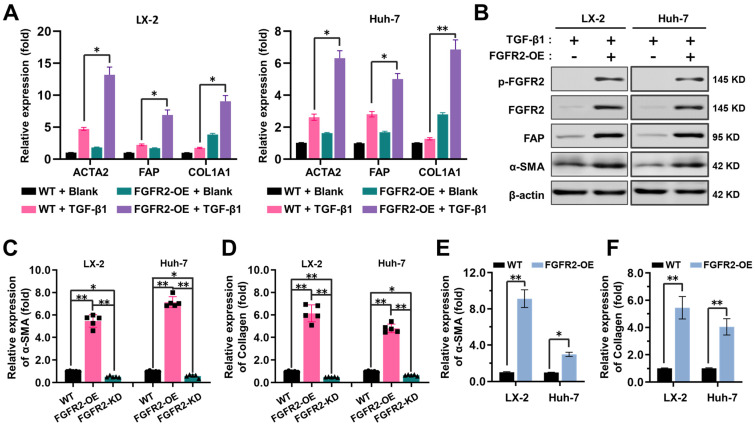
FGFR2 drives the process of liver fibrosis. (**A**) The expression of liver fibrosis markers (ACTA2, fibroblast activation protein alpha (FAP), alpha-1 type I collagen (COL1A1)) was measured by qPCR. The samples were divided into four groups according to whether cells were induced with or without TGF-β and whether cells were overexpressed by FGFR2. (**B**) The expression of markers in wild-type and FGFR2-OE cell lines under TGF-β induction was analyzed by Western blot analysis. (**C**) The expression levels of α-SMA and (**D**) collagen secretion in LX-2 and Huh-7 were evaluated, when FGFR2 was overexpressed or knocked down upon equal TGF-β induction. (**E**) Cells were collected after 48 h of co-culture, and the expression of α-SMA and (**F**) type I collagen in the lower layer cells was determined by ELISA. The results are marked as significant “*” when *p* < 0.05, “**” when *p* < 0.01, and not significant (ns) if *p* ≥ 0.05.

In order to verify the regulatory role of FGFR2 in the development of liver fibrosis, two cell lines were established, i.e., the FGFR2 overexpressing HSC cell line (LX-2) and the liver cell line (Huh-7), and the sensitivity changes induced by TGF-β were compared between the two cell lines. The qPCR and Western blot results showed that when FGFR2 was overexpressed, the expression of biological markers associated with liver fibrosis, including ACTA2 (coding α-SMA), fibroblast activation protein alpha (FAP), and alpha-1 type I collagen (COL1A1), was significantly upregulated in both HSC cells and liver cells (Figure 2A,B). Subsequently, through comparison with the FGFR2 knockdown cell line (FGFR2-KD, Supplementary Figure S1A), it was found that, upon equal TGF-β induction, the α-SMA in the cells (Figure 2C) and the secreted collagen (Figure 2D) level increased with the upregulation of FGFR2, while these parameters decreased following FGFR2 knockdown. This indicates that FGFR2 knockdown reduced the cells’ sensitivity to TGF-β induction, thereby maintaining lower levels of α-SMA expression and collagen secretion. Interestingly, based on the co-culture model, it was discovered that FGFR2-upregulated cells can deliver more activating signals to wild-type cells, thereby promoting the activation of wild-type cells (Supplementary Figure S1B, Figure 2E,F). The above results demonstrate that FGFR2 promotes the progression of liver fibrosis, and the inactivation of FGFR2 can reverse this promotion effect.

### 2.3. Cynaroside (CYN) Blocks the Activity of FGFR2

**Figure 3:**
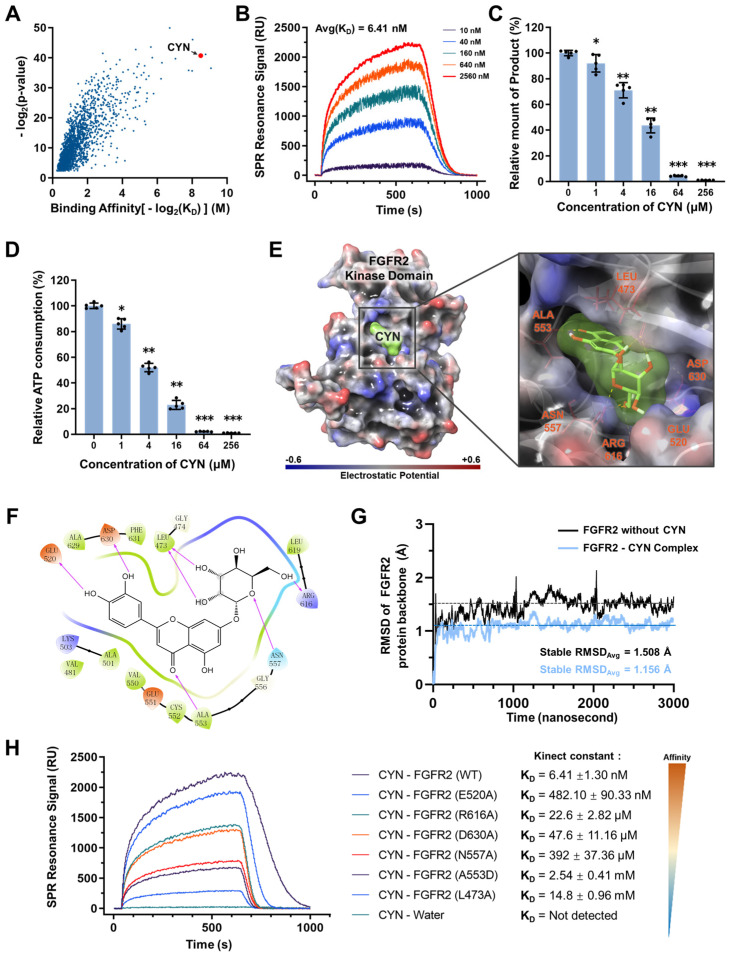
CYN blocks the activity of FGFR2. (**A**) A total of 2081 natural compounds were screened through high-throughput affinity analysis chips, resulting in the identification of compounds capable of binding to the FGFR2 kinase domain. The affinity and *p*-value of each compound were represented, and the candidate compound CYN was located in the high-affinity region. (**B**) The binding signal of FGFR2 and CYN was analyzed using surface plasmon resonance (SPR) technology at five different concentrations, and the affinity constant K_D_ was calculated by fitting the curve algorithm. (**C**) An in vitro FGFR2 catalytic system was established, and the kinase activity was compared by measuring the phosphorylation product generation and (**D**) ATP consumption during the catalytic process. A series of concentration gradients were set up to evaluate the correlation between the inhibitory effect of CYN and its concentration. (**E**) Computational docking was utilized to predict the binding site of CYN on FGFR2. The surface charges on the model are depicted in blue (negative) to red (positive), and the appearance of the compound CYN is represented in green. Noncovalent amino acids that provide binding for the compound are marked in red on the right figure. (**F**) The diagram depicts the binding of the CYN and FGFR2 amino acid residues. (**G**) Molecular dynamics (MD)simulations were conducted to compare the stability of FGFR2 protein and FGFR2-CYN complex in aqueous environments. The mean value of the root-mean-square deviation (RMSD)at the plateau period is marked by a dotted line. (**H**) SPR was used to measure the affinity between the CYN and FGFR2 proteins with single-point mutations. The affinity is ranked from strong to weak in the legend, and the right-side values represent the corresponding K_D_ values. A higher K_D_ value represents weaker binding of the mutated FGFR2 protein with CYN, indicating that CYN’s binding is more dependent on the mutated amino acid. The results are marked as significant “*” when *p* < 0.05, “**” when *p* < 0.01, “***” when *p* < 0.001, and not significant (ns) if *p* ≥ 0.05.

With the aim of discovering a therapeutic agent to inhibit FGFR2 and mitigate liver fibrosis, high-throughput affinity screening based on surface plasmon resonance (SPR) sensor chips were carried out with a natural compound library. This identified several compounds with the potential to bind to the FGFR2 kinase domain, among which CYN was found to be a high-affinity compound (Figure 3A) with an affinity constant (K_D_) of 6.41 nM (Figure 3B). In addition, based on the monitoring of the generation of phosphorylation products and ATP consumption in an in vitro catalytic system, the inhibitory effect of CYN on FGFR2 activity increased progressively as its concentration increased (Figure 3C,D). The computational docking results showed that CYN forms a noncovalent bond with six surrounding amino acid residues at the catalytic pocket of FGFR2 (Figure 3E,F). The stability analysis of the molecular dynamics (MD) simulations revealed that CYN formed a highly stable complex with FGFR2 in a liquid environment, with a root-mean-square deviation (RMSD) of 1.156 Å between CYN and the α-carbon backbone of FGFR2. In contrast, the RMSD of FGFR2 without CYN binding was 1.508 Å (Figure 3G). These results demonstrate that the stability of the FGFR2-CYN complex is higher than that of the FGFR2 protein alone, indicating the stable formation of the FGFR2-CYN complex. We further performed a comparative analysis of the root-mean-square fluctuation (RMSF) of amino acids in the vicinity of the FGFR2 binding site in the presence and absence of CYN (Supplementary Figure S1C,D). The results revealed that the absence of CYN led to higher fluctuations of amino acids in the binding groove, which can be attributed to their exposure to the surrounding liquid environment.

To further confirm the substructure binding site of CYN on FGFR2, we analyzed the binding capacity of CYN to various single-point mutant forms of FGFR2. The results indicated a significant decrease in the binding capacity between FGFR2 and CYN after the mutation of specific amino acids, particularly L473A and A553D (Figure 3H). This demonstrates that the binding of CYN to FGFR2 is dependent on these two amino acids and confirms that the binding sites of CYN on the FGFR2 protein are located in the catalytic pocket.

### 2.4. CYN Blocks the Activation and Development of Liver Fibrosis

**Figure 4:**
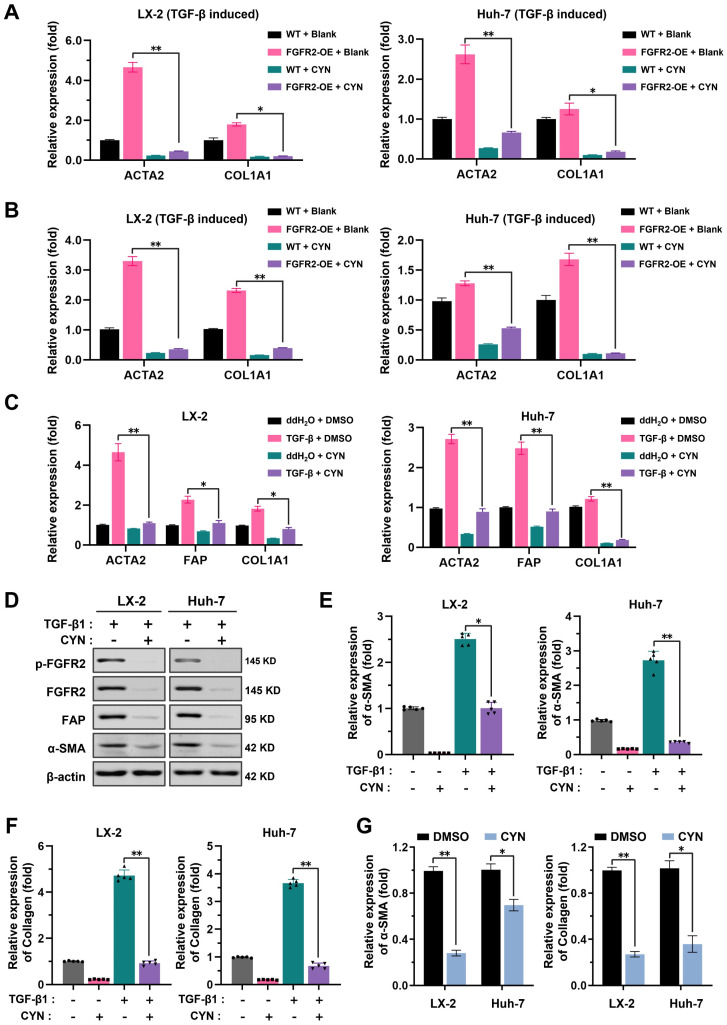
CYN blocks the activation and development of liver fibrosis in vitro. (**A**) Evaluation of the inhibitory effect of CYN on the fibrosis-promoting effect of FGFR2. The fibrotic transformation of wild-type and FGFR2-OE LX-2 cells and Huh-7 cells was induced through TGF-β activation, followed by an intervention with CYN for the relevant groups. The expression changes of the fibrosis markers ACTA2 and COL1A1 were analyzed by qPCR. (**B**) Wild-type cell lines were employed in the aforementioned experiments, and the activation of FGFR2 was triggered by supplementation with the exogenous basic fibroblast growth factor (bFGF) factor. (**C**) Analysis of the extent of antagonism of CYN towards TGF-β Signaling. Activation induction models were established by adding or not adding TGF-β to the cell culture environment with or without the CYN intervention, and the expression of liver fibrosis markers was determined by qPCR, (**D**) Western blot, and (**E**,**F**) ELISA analyses. (**G**) A co-culture model was used to evaluate the blocking effect of CYN on liver fibrosis activation of signaling transmission. The activation intensity of lower-layer wild-type cells was measured and compared using α-SMA expression and collagen secretion. The results are marked as significant “*” when *p* < 0.05, “**” when *p* < 0.01, and not significant (ns) if *p* ≥ 0.05.

The study then aimed to evaluate the inhibitory effect of CYN on the fibrosis-promoting effect of FGFR2. The presence of CYN was found to reduce the sensitivity of wild-type and FGFR2-OE LX-2 cells and Huh-7 cells to TGF-β, leading to a reduction in the expression of fibrosis-related markers (Figure 4A). CYN was also observed to block the overactivation of FGFR2 induced by basic fibroblast growth factor (bFGF), an FGFR2 ligand that transmits signals between cells (Figure 4B). Furthermore, results from the qPCR, Western blot, and ELISA collectively demonstrated that the presence of CYN reversed the increased expression of fibrosis markers caused by TGF-β (Figure 4C,D) and reduced the levels of α-SMA expression (Figure 4E). and collagen secretion (Figure 4F). The co-culture model also showed that CYN weakened the activation of lower-layer cells in response to upper-layer activated cells (Figure 4G). In conclusion, the presence of CYN reduces the sensitivity of LX-2 cells and Huh-7 cells to TGF-β, and this inhibitory effect is also suitable for FGFR2 hyperactivation cases.

### 2.5. CYN Attenuates the Progression of Liver Fibrosis In Vivo

**Figure 5:**
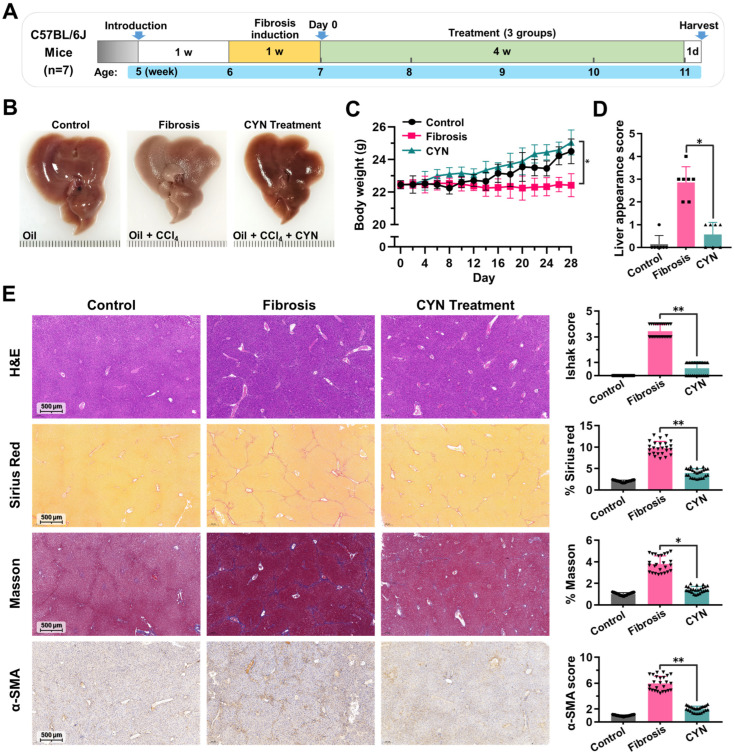
CYN attenuates the progression of CCl_4_-induced liver fibrosis. (**A**) Schematic of the animal experiment. (**B**) Liver appearance at the time of sample collection. (**C**) The trend of mouse weight over time where Day 0 is the initial day of treatment. (**D**) Liver appearance score. (**E**) Results for liver sections stained with hematoxylin and eosin (H&E), Sirius Red, Masson, and immunohistochemistry (IHC)for α-SMA. The statistics are based on quantitative data from 25 random fields of view for all animals in this group. The results are marked as significant “*” when *p* < 0.05, “**” when *p* < 0.01, and not significant (ns) if *p* ≥ 0.05.

The therapeutic effect of CYN on liver fibrosis was assessed in two mouse models: the CCl_4_-induced liver fibrosis model and the high-fat-diet-induced NASH model. The animal models and dosing regimens used for the former are depicted in Figure 5A and Supplementary Figure S2A. In the liver tissues of the mice, it was observed that the fibrosis group displayed fibrosis-like changes, whereas the CYN-treated group exhibited milder symptoms (Figure 5B). Additionally, the CYN-treated mice demonstrated more normal bodyweight trends (Figure 5C), liver appearances (Figure 5D), and serum alanine transaminase (ALT) and aspartate transaminase (AST) levels (Supplementary Figure S2B,C) compared with the fibrosis group. These findings suggest that the CYN-treated mice had a lower degree of liver damage compared to the fibrosis group. Subsequently, liver tissue samples were sectioned and stained, which revealed that the liver tissues from the CYN-treated group possessed more normal morphologies, a lower percentage of Sirius red staining, reduced collagen expression, and decreased α-SMA expression (Figure 5E). These results suggest that CYN has the potential to delay the development of carbon-tetrachloride-induced liver fibrosis.

**Figure 6:**
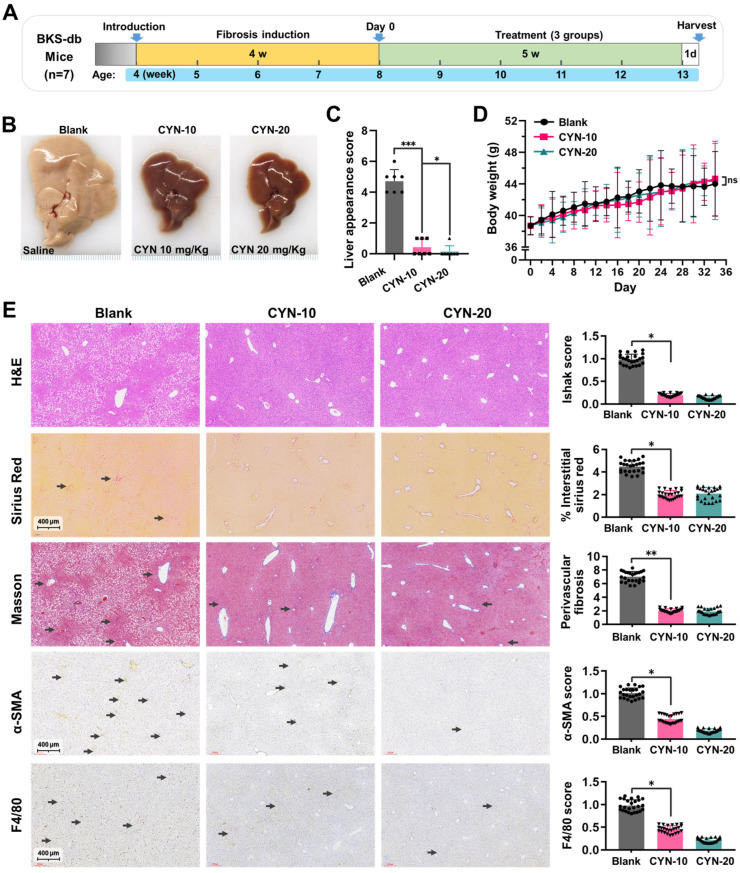
CYN attenuates the progression of NASH-background liver fibrosis. (**A**) Schematic of the animal experiment. (**B**) Liver appearance at the time of sample collection. (**C**) Liver appearance score. (**D**) The mouse weight trend over time. (**E**) Results of liver sections stained with H&E, Sirius Red, Masson, and IHC for α-SMA and EGF module-containing mucin-like receptor (F4/80). The statistics are based on quantitative data from 25 random fields of view for all animals in this group. The arrows indicate regions with pronounced staining. The results are marked as significant “*” when *p* < 0.05, “**” when *p* < 0.01, “***” when *p* < 0.001, and not significant (ns) if *p* ≥ 0.05.

The high-fat diet NASH animal model was also utilized to evaluate the therapeutic effect of CYN (Figure 6A, Supplementary Figure S2D). Consistent with the above findings, the mice in the CYN-treated group demonstrated more normal liver morphologies (Figure 6B,C). Meanwhile, CYN treatment did not influence the bodyweights (Figure 6D), food intakes (Supplementary Figure S2E), or blood glucose contents (Supplementary Figure S2F) of the mice. The staining of liver sections indicated that the CYN-treated group showed less steatosis, a lower percentage of sirius red staining in interstitial liver tissue, reduced perivascular collagen expression, and decreased α-SMA expression compared with the blank group. Furthermore, EGF module-containing mucin-like receptor (F4/80) staining revealed that CYN treatment resulted in a milder intrahepatic inflammatory environment. In addition, it was found that the administration of CYN at a dose of 20 mg/kg resulted in improved outcomes compared to a dose of 10 mg/kg. These results suggest that CYN exhibits a protective effect on the liver in response to NASH-induced liver fibrosis. In conclusion, these findings suggest that CYN has the potential to attenuate the progression of liver fibrosis in vivo and could be a promising option for the clinical treatment of liver fibrosis (Figure 6E).

## 3. Discussion

In past studies, FGFR2 has shown the potential to be used as a target for the treatment of liver fibrosis [17,19,21,22,23,24]. Current evidence suggests that FGFR2 may be a promising target [25,26,27], but more research is needed to determine the efficacy of FGFR2 as a target for liver fibrosis treatment as well as to fully understand the mechanisms behind its involvement in TGF-β signaling. In this study, the correlation between FGFR2 expression and liver fibrosis development was demonstrated through data mining. The results from the cellular assays indicated heightened responsiveness to TGF-β signaling in stellate cells and hepatocytes with FGFR2 overexpression. The high expression of FGFR2 was also shown to facilitate the intercellular transmission of signals that activate stellate cells. In response, a small molecule compound, CYN, which targets the intracellular kinase domain of FGFR2, was screened from the compound library. The potential of CYN was validated through simulated docking, MD analysis, binding affinity analysis, and single-point mutation validation. These analyses demonstrated the ability of CYN to block the catalytic pocket of FGFR2. The results of the cellular assays revealed that CYN can inhibit FGFR2 hyperactivation resulting from its overexpression and excessive bFGF and reduce HSC cell activation and collagen secretion in hepatocytes. The animal experiments showed that CYN treatment reduced liver fibrosis during fibrosis formation. These findings suggest that CYN may have a preventative effect on liver fibrosis formation and could offer a potential solution for the clinical treatment of liver fibrosis.

To predict the binding mode of CYN to FGFR2, we performed docking simulations to identify the most stable region of CYN bound to FGFR2. The results showed that CYN is connected to six amino acid residues through seven hydrogen bonds. During the formation of these hydrogen bonds, five hydroxyl groups of CYN act as H-donors, and one carbonyl oxygen and one ether oxygen act as H-acceptors. Alanine scanning based on single point mutation and SPR assays demonstrated that CYN binding to FGFR2 is highly dependent on residues 473L and 553A. By inspecting the structural diagram, we found that FGFR2’s 473L is close to amino acid 466Y, which is involved in FGFR2 phosphorylation activation regulation [28,29]. This may prevent 466Y from being self-phosphorylated, thus blocking activation of the FGFR2 kinase domain. In addition, compound binding covered the ATP binding site from 487 to 495, blocking contact between this region and the substrate, which may also be a cause of FGFR2 kinase inactivation. Residue 553A is a neutral residue located between two catalytic domains [29]. Although there is currently no report on the function of 553A, we speculate, according to RMSF analysis (Supplementary Figure S1C,D), that it may participate in connecting the two domains and provide a certain degree of freedom for the two catalytic domains to undergo appropriate conformational changes when catalyzing different substrates. CYN binds to 553A and 557N through hydrogen bonding, fixing the originally free linker and increasing the difficulty of conformational changes at the FGFR2 catalytic center.

Currently, there are several clinical treatment options available for liver fibrosis, but few of them directly target TGF-β signaling and collagen accumulation [4,30]. These clinical options include antiviral drugs [31,32], such as interferon and ribavirin, for viral hepatitis; immunosuppressive agents [33], such as azathioprine and mycophenolate mofetil, for inhibiting the immune system; corticosteroids [34,35], including prednisone, for reducing inflammation; and lipid-reducing drugs [36,37], such as simvastatin and its derivatives. The principle behind these treatment options is to indirectly reduce the pathogenesis or damage and slow down the progression of liver fibrosis. However, the pathogenesis of liver fibrosis is complex and multifactorial, involving the interaction of multiple factors. Inhibiting the hepatic response to TGF-β signaling and reducing collagen accumulation may represent more direct approaches. Only a few drugs have been reported to have direct anti-fibrotic capabilities, such as pirfenidone [38,39], which underwent clinical trials in 2005. The discovery of CYN offers a novel treatment approach that inhibits FGFR2 to desensitize HSCs and hepatocytes to TGF-β signaling, thereby reducing collagen expression. This approach provides additional options for the clinical treatment of liver fibrosis.

CYN, discovered in this study, could also be used as a targeted therapeutic approach, as it can inhibit FGFR2, weakening the activation of stellate cells and the production of collagen. Recent evidence suggests that sorafenib, an anticancer drug targeting FGFR2, may have the potential to treat liver fibrosis [40,41,42]. However, recent studies have shown that the long-term use of sorafenib may result in vascular endothelial damage and liver injury or even liver failure [43,44]. These adverse effects may result from excessive inhibition or multiple target effects.

CYN overcomes this limitation, as experimental data show that it produced no acute toxicity effects, such as weight loss or liver dysfunction, after 35 days of administration. CYN does not affect food consumption or blood glucose levels, suggesting that it has no impact on metabolism. Compared to sorafenib, CYN has a similar structure with a sugar ring substitution and more hydrophilic groups. This structural difference may result in a shorter half-life and weaker receptor binding affinity, but it improves CYN’s safety profile and enables its long-term use for preventive actions. At the molecular level, drug efficacy is largely determined by target specificity. During high-throughput screening, CYN was identified as the most promising treatment candidate, despite not having the strongest affinity to FGFR2. This was due to the elimination of drugs with broad toxicity through toxicity control points, whereas CYN exhibited low toxicity and a high rate of FGFR2 inhibition. The dose of the drug plays a crucial role in its efficacy. The study administered a dose of 10–20 mg/kg to mice, and the results showed that there was better antagonism against liver fibrosis at higher doses. However, a balance among efficacy, safety, and cost is yet to be found, and this will be the focus of future research. After pharmacological and pharmacogenetic refinement, CYN has the potential to be used as a new treatment for liver fibrosis.

## 4. Materials and Methods

### 4.1. Data Mining and Clinical Samples

Clinical datasets for FGFR2 data mining were collected from the NCBI Gene Expression Omnibus (GEO) database. Five gene expression profiles were used, including human HBV infection (GSE38941 [45]), alcohol abuse (GSE28619 [46]), NASH (GSE48452 [47]), and CCl_4_-induced liver fibrosis in mice (GSE152329 [48], GSE98577). The expression of FGFR2 was also analyzed before and after liver fibrosis treatment (GSE175448 [49]). The tissue samples were anonymously collected from the First Affiliated Hospital of Jinan University and donor information was recorded but kept confidential. The tissue was anonymously donated from the sample center of the First Affiliated Hospital of Jinan University, and donor information was recorded but kept confidential.

### 4.2. Molecular Evaluation

The candidate compound, CYN, was obtained from Selleck Co., Ltd. (Houston, TX, USA). The qPCR analysis was performed using TaKaRa’s ExTaq (RR001Q) with the primers listed in Supplementary Table S1 according to the official manual. The gene expression levels were calculated and compared using ΔΔCt values. The Western blot and IHC analyses were performed using primary antibodies against FGFR2 (23328S, CST, Danvers, MA, USA), phospho-FGFRs (AF3285, R&D, Minneapolis, MN, USA), α-SMA (ab7817, Abcam, Waltham, MA, USA), FAP (AF3715, R&D, Minneapolis, MN, USA), and β-actin (MAB8929, R&D, Minneapolis, MN, USA). The protein content was confirmed by grayscale analysis of the bands. IHC analysis was conducted by counting the number of stained cells using ImageJ. Recombinant proteins of FGFR2 (100550-MM07) and bFGF (10014-HNAE) were obtained from Sinobiological, Beijing, China. α-SMA was quantified with the Human alpha SMA ELISA Kit (Abcam, Waltham, MA, USA), while the collagen content was quantified with the Human Type 1 Collagen ELISA Kit (Abmart, Shanghai, China). FGFR2 phosphokinase activity was assessed through the detection of ATP consumption and the phosphorylated substrate product using the Kinase-Lumi luminescent kinase assay kit (Beyotime, Shanghai, China) and the universal tyrosine kinase assay kit (TAKARA, Shiga, Japan). All experiments using commercial kits were performed according to the manufacturer’s instructions.

### 4.3. Cell Evaluation Procedure

The human hepatic stellate cell line (LX-2) and human hepatocellular carcinoma line (Huh-7) were sourced from Procell (Wuhan, China) and cultured in DMEM with 10% FBS in a 5% CO_2_ incubator at 37 °C. The cell identity was verified by short tandem repeat (STR) analysis, and the mycoplasma status was confirmed with monthly noncontaminated testing. The coculture was performed using Transwell chambers with 0.8 μm pores (Thermo Fisher, Waltham, MA, USA) and Matrigel as a scaffold (BD, Franklin Lakes, NJ, USA). FGFR2 overexpression and knockdown were achieved via lentiviral transduction. FGFR2-OE and vehicle lentiviruses were created by inserting genes into the pCDH-CMV-MCS-EF1-Puro and pLP1, pLP2, and pLP-VSVG packaging system (System Biosciences, Palo Alto, CA, USA). FGFR2-KD and mimic lentiviruses were made by inserting three shRNA sequences (Supplementary Table S2) or nonsense RNAi control sequences into pCDH-U6-shRNA-EF1-Puro. Transfection and selection using puromycin were performed according to the manufacturer’s protocol.

### 4.4. High Throughput Screening of Drugs and Binding Affinity Verification

Label-free high-throughput drug screening was conducted using the bScreen LB 991 Label-free Microarray System and label-free photo-cross-linker sensor chips (Betterways Inc., Guangzhou, China). The bioactive compound library (L1700, version 2018, 2081 compounds, Selleck Inc., Houston, TX, USA) was printed on biochips with a BioDot™ AD-1520 Array Printer (BIODOT Inc., Irvine, CA, USA), and the affinity between each compound and the FGFR2 kinase domain was measured by SPR technology (Betterways Inc., Guangzhou, China). Toxicity screening was then performed with a discard threshold of IC_50_ < 10 μM in LX-2 cells.

### 4.5. In Silico Docking and MD Simulation

In silico docking was executed through the application of Schrödinger’s Discovery Suite (Schrödinger, version 2022-1) and KNIME Workflow (KNIME Analytics Platform, version 4.3.3). The FGFR2 kinase domain structure (PDB ID: 7OZY [50]) was optimized using automated protein preparation, which involved side chain repair, H-bond optimization, protein minimization, and removal of excess water molecules. A binding site characterization workflow was employed to identify all possible ligand binding sites, with a minimum reportable site of 15 Å and cropping of sites within 4 Å. The receptor grid generation algorithm was used to fill these binding sites with a grid using default parameters. In the ligand-docking stage, the protein model and CYN structure were utilized to perform extra-precision (XP) docking, and the compound-protein complex with the lowest energy was selected as the most stable conformer. Binding free energy was calculated using specific algorithms [51,52,53]. MMGBSA was used to analyze bond properties with parameters including force field = OPLS4 and solvation model = VSGB. The stability of the docking complex was confirmed through 3000 ns MD simulations using Desmond software (DE Shaw Research, version 2023-1) with calculation parameters including solvent model = SPC water model, box shape = ortho-rhombic with 15 Å beyond the protein edge, ionic additives = 0.15 M NaCl, ensemble class = NPT, initial temperature = 310.15 Kelvin, pressure = 1.01325 bar, and force field = OPLS4. Trajectory analysis was performed automatically using Desmond software [54], and binding stability was evaluated by computing the RMSD and the conformational changes were assessed using RMSF per residue.

### 4.6. Animal Experiments

The animal study was approved by the Institute of Laboratory Animal Science at Jinan University and carried out following the animal welfare guidelines. Four-week-old male C57BL/6J mice (Cyagen Biosciences Inc., Guangzhou, China) were acclimatized for 1 week before being utilized in the establishment of the CCl_4_-induced fibrosis model. Male BKS-db mice, also 4 weeks old (Jingle Biotech, Guangzhou, China), were acclimatized and fed a high-fat diet for 4 weeks to establish the NASH model. The feed provided to the mice (SYGR01, Yushu Biotech, Shanghai, China) had a caloric value of 4.13 kcal/g and was made freely available for consumption by the mice. The experimental design and treatment plan are illustrated in Figure 5A and Figure 6A. Bodyweight was recorded daily. A histological analysis was performed using H&E, Sirius Red, Masson, and immunohistochemical staining (Beyotime, Shanghai, China, and Sangon, Shanghai, China) in accordance with the manufacturer’s instructions.

### 4.7. Statistical Analysis

The data from each experimental step were analyzed using GraphPad Prism software (version 9.1). The results were presented as the mean ± standard deviation (SD). The statistical significance of the differences was evaluated using the two-tailed Student’s *t*-test or one-way ANOVA. Multiple measurements were performed with a minimum of three replicates, unless otherwise specified. The statistical significance was determined based on the *p*-value. The results are marked as significant “*” when *p* < 0.05, “**” when *p* < 0.01, and not significant (ns) if *p* ≥ 0.05.

## 5. Conclusions

In conclusion, this study has shown that high expression of FGFR2 is correlated with liver fibrosis and that FGFR2 drives the process of liver fibrosis. Furthermore, CYN was identified as a natural compound that can block the activity of FGFR2 by forming a stable complex in the catalytic pocket of FGFR2. The inhibitory effect of CYN on the fibrosis-promoting effect of FGFR2 was also demonstrated, as CYN reduced the sensitivity of cells to TGF-β induction and mitigated the development of liver fibrosis. Therefore, CYN may be a promising therapeutic agent for the treatment of liver fibrosis, and further research is needed to explore its potential clinical application.

## Data Availability

Data is contained within the article and supplementary material.

## Supplementary Materials

The following supporting information can be downloaded at: https://www.mdpi.com/article/10.3390/ph16040548/s1, Figure S1: CYN blocks the liver fibrosis promoting activity of FGFR2; Figure S2: CYN attenuates the progression of liver fibrosis; Table S1: Primer List; Table S2: shRNA List.
