# Supplementary material for: Inhibition of FGFR2 Signaling by Cynaroside Attenuates Liver Fibrosis
Source: Pharmaceuticals (Basel). 2023 Apr 6;16(4):548. doi: 10.3390/ph16040548 (PMC10145669; doi:10.3390/ph16040548)
Supplement: Supplementary file 1 [file pharmaceuticals-16-00548-s001.zip › sFigures.pdf]

Supplementary Figures

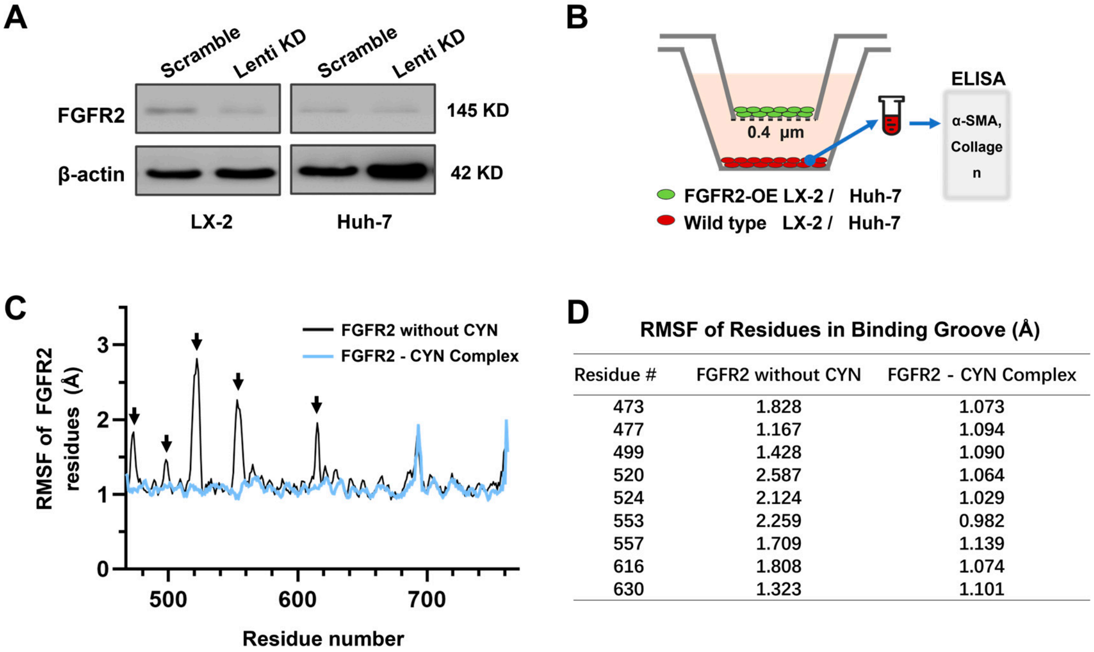

Residue #	FGFR2 without CYN	FGFR2 - CYN Complex
473	1.828	1.073
477	1.167	1.094
499	1.428	1.090
520	2.587	1.064
524	2.124	1.029
553	2.259	0.982
557	1.709	1.139
616	1.808	1.074
630	1.323	1.101

Supplementary Figure S1. CYN blocks the liver fibrosis promoting activity of FGFR2. (A) The FGFR2 expression levels in knock down cell lines were determined by Western blot analysis. Scramble: transfected with lentivirus containing scramble sequences; Lenti KD: transfected with lentivirus containing shRNA coding sequences targeting FGFR2. (B) A co-culture model was established, in which overexpressed cells were cultured in the upper chamber and wild-type cells were cultured in the lower chamber separated by a 0.8 μm filter to impede cell permeation. (C) A comparative analysis was conducted to assess the root-mean-square fluctuation (RMSF) of amino acids surrounding the FGFR2 binding site in the presence and absence of CYN. The region indicated by the arrows represents residues with significant RMSF difference. (D) The amino acids involved in CYN binding and their respective RMSF values obtained from molecular dynamics simulations are presented in the statistical table.

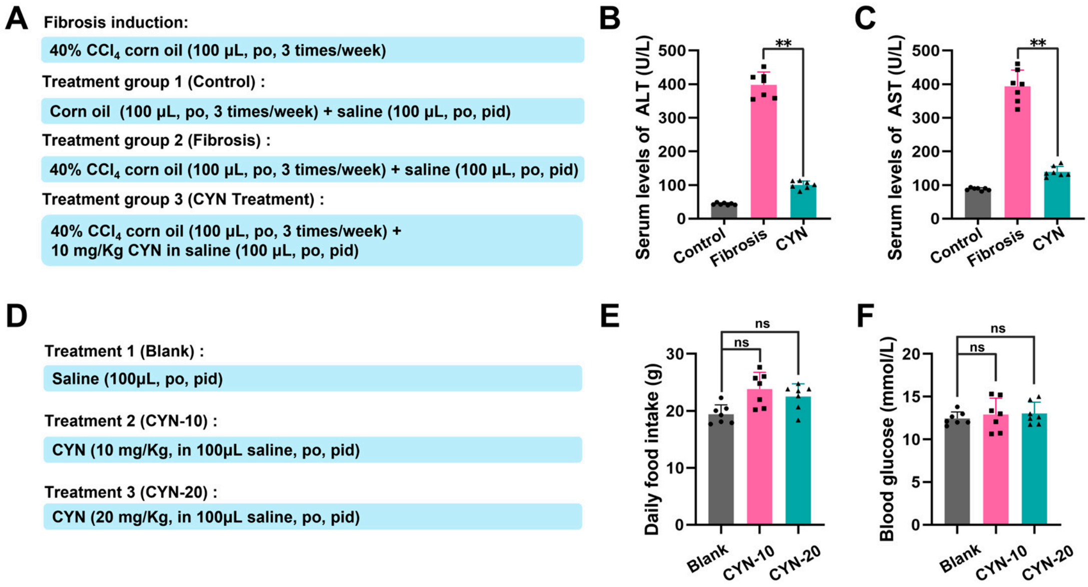

Supplementary Figure S2. CYN attenuates the progression of liver fibrosis. (A) Fibrosis induction phase and administration protocol for each treatment group in CCl4-induced liver fibrosis. (B) Serum ALT levels. (C) Serum AST levels. (D) Fibrosis induction phase and administration protocol for each treatment group in NASH-background liver fibrosis. (E) Average daily food intake from Day 0 to Day 35. (F) The average blood glucose level at Day 35.
